# Depression and Anxiety Among Women in Lesotho: A Multilevel Analysis of the 2023–2024 LSDHS

**DOI:** 10.1155/da/1572451

**Published:** 2026-05-21

**Authors:** Helen Lamesgin Endalew, Astewil Moges Bazezew, Eniyew Assimie Alemu, Enyew Getaneh Mekonen, Berihun Agegn Mengistie, Gebreeyesus Abera Zeleke, Desalegn Getachew Ayele, Amlaku Nigusie Yirsaw, Getie Mihret Aragaw, Alemken Eyayu Abuhay, Degsew Ewunetie Anteneh, Gebeyehu Lakew

**Affiliations:** ^1^ Department of Surgical Nursing, School of Nursing, College of Medicine and Health Sciences, University of Gondar, Gondar, Ethiopia, uog.edu.et; ^2^ Department of Anesthesia, College of Medicine and Health Science, University of Gondar, Gondar, Ethiopia, uog.edu.et; ^3^ Department of General Midwifery, School of Midwifery, College of Medicine and Health Sciences, University of Gondar, Gondar, Ethiopia, uog.edu.et; ^4^ Department of Health Promotion and Health Behavior, Institute of Public Health, College of Medicine and Health Sciences, University of Gondar, Gondar, Ethiopia, uog.edu.et; ^5^ Department of Clinical Midwifery, College of Medicine and Health Sciences, University of Gondar Comprehensive Specialized Referral Hospital, Gondar, Ethiopia, uog.edu.et

**Keywords:** anxiety, depression, Lesotho, multilevel analysis, reproductive age

## Abstract

**Background:**

Women’s mental health significantly influences families, communities, and national development. In low‐resource settings like Lesotho, factors such as chronic illness, financial barriers, and limited access to care contribute to the burden of mental health conditions. However, population‐level evidence on depression and anxiety among reproductive age women remains limited.

**Methods:**

A cross‐sectional multilevel analysis was conducted using data from the 2023–2024 Lesotho Demographic and Health Survey (LSDHS). A weighted sample of 3265 women aged 15–49 years was included. Outcomes were defined as physician‐diagnosed depression, Patient Health Questionnaire (PHQ)‐based depressive symptoms, and self‐reported anxiety. Multilevel multivariable logistic regression models were fitted separately for each outcome to identify associated factors while accounting for the hierarchical structure of the data.

**Results:**

The prevalence of self‐reported physician‐diagnosed depression was 10.62%, PHQ‐based depressive symptoms 5.37%, and self‐reported physician‐diagnosed anxiety was 9.07%. Chronic illness was significantly associated with all three outcomes. Financial barriers to healthcare were associated with self‐reported physician‐diagnosed depression and PHQ‐based depressive symptoms, while higher parity and younger age were associated with PHQ‐based depressive symptoms. Smoking and lack of media exposure were associated with higher odds of self‐reported physician‐diagnosed anxiety. Regional disparities were observed for self‐reported physician‐diagnosed depression and anxiety.

**Conclusions:**

Mental health problems remain a significant concern among women in Lesotho. Both shared and outcome‐specific factors were identified, with chronic illness emerging as a consistent determinant. These findings highlight the need for integrated and targeted mental health interventions, particularly within primary healthcare and chronic disease management, alongside efforts to improve access to information and reduce regional inequalities.

## 1. Introduction

Globally, depression and anxiety rank among the most common mental health disorders, substantially contributing to disability and disease burden [1]. Depression manifests as a persistent low mood, loss of interest, feelings of guilt or worthlessness, fatigue, and suicidal thoughts [2]. Anxiety disorders are characterized by excessive worry, fear, and restlessness, often accompanied by physical symptoms such as an increased heart rate and muscle tension. According to the DSM‐V, anxiety disorders include generalized anxiety disorder, panic disorder, social anxiety disorder, and phobias [3]. These conditions frequently overlap, sharing symptoms such as difficulty concentrating, disturbed sleep, and irritability [4].

Worldwide, more than 300 million people are affected by depression, and approximately 3.6% experience anxiety disorders, with women being disproportionately affected [5–7]. The combined impact of these disorders reduces productivity, worsens the quality of life, and increases healthcare costs. In low‐ and middle‐income countries (LMICs), limited mental health resources, low awareness, and sociocultural stigma exacerbate the burden [8–10].

The prevalence of depression and anxiety varies across countries, reflecting differences in social, cultural, and economic contexts. In China, population‐based studies report a 12‐month prevalence of depression at approximately 3.6% and anxiety disorders at 5.0% among adults [11–13]. In Australia, the 12‐month prevalence of major depressive disorder is estimated at around 10% [14]. In Ghana, anxiety symptoms affect roughly 24.2% of the population [15], while among Somali and Kenyan refugees, depression and anxiety comorbidity can reach as high as 33.6%. In Ethiopia, depression is reported at approximately 10% [16].

These variations underscore the importance of generating context‐specific mental health data to inform effective national mental health strategies.

Multiple sociodemographic and behavioral factors, including age, marital status, socioeconomic status, chronic illness, and alcohol use, are consistently associated with depression and anxiety [6, 17]. Among women, biological factors such as hormonal changes related to menstruation, pregnancy, and menopause may interact with social stressors, including gender‐based violence, caregiving responsibilities, and economic hardship, thereby increasing vulnerability to mental health problems [18]. In LMICs, limited access to education, employment opportunities, and healthcare services further exacerbates these risks [19, 20].

In Lesotho, mental health services remain scarce, with few specialized facilities and a critical shortage of trained professionals. Existing services are primarily hospital‐based, particularly at Mohlomi Mental Hospital in Maseru, with limited community‐level outreach. Stigma, poor mental health literacy, and low service coverage contribute to substantial treatment gaps. These barriers highlight the need for population‐based evidence to guide equitable and decentralized mental health planning [21].

The inclusion of mental health indicators in the 2023–2024 Lesotho Demographic and Health Survey (LSDHS) provides a valuable opportunity to examine depression and anxiety among women using nationally representative data. Using this dataset, the present study estimates the prevalence of physician‐diagnosed depression, Patient Health Questionnaire (PHQ)‐based depressive symptoms, and anxiety, and identifies associated individual‐, household‐, and community‐level factors among women aged 15–49 years. This analysis aims to generate evidence to inform targeted and context‐specific mental health interventions in Lesotho.

### 1.1. Theoretical Framework of the Study

**Figure 1:**
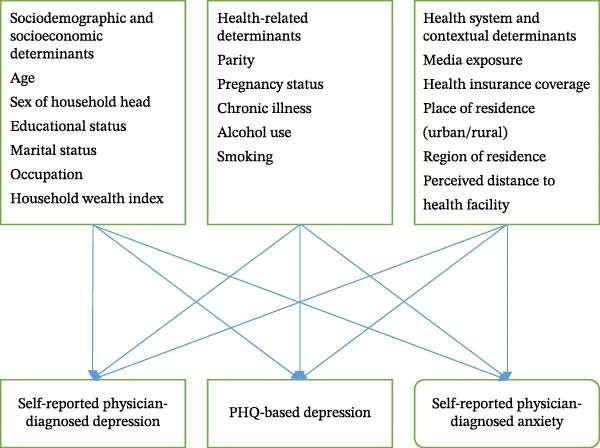
Conceptual framework for depression and anxiety among women in Lesotho, 2023–2024 LSDHS.

This study was guided by the World Health Organization (WHO) Social Determinants of Health (SDH) framework [22] (Figure 1). The SDH framework explains that mental health outcomes, including depression and anxiety, are shaped by the social and economic conditions in which individuals are born, live, and age. These determinants operate at multiple levels, including individual, household, and contextual levels.

In this study, individual factors (such as age, education, marital status, and parity), household factors (such as the wealth index and sex of household head), and contextual factors (such as residence and media exposure) were considered as key determinants of depression and anxiety among reproductive‐aged women in Lesotho. The SDH framework posits that inequalities in these social conditions influence exposure to risk and access to protective resources, thereby contributing to differences in mental health outcomes across populations.

## 2. Methods and Materials

### 2.1. Study Design, Setting, and Participants

A cross‐sectional, community‐based multilevel secondary data analysis was conducted using data from the 2023–2024 LSDHS, which is nationally representative and was implemented across all 10 districts of Lesotho. The survey employed a two‐stage stratified sampling technique: enumeration areas (EAs) were first selected using probability proportional to size, followed by the systematic selection of households. All women aged 15–49 years who were usual residents or visitors the night before the survey were eligible for an interview.

The data have a hierarchical structure, with individuals (level 1) nested within households (level 2) and households nested within clusters (level 3). A total of 6413 women were interviewed. For this study, we used the individual recode (IR) dataset. Of the total, 3116 women were excluded due to missing data on depression, anxiety, and PHQ‐based depressive symptoms, which were collected only in a subsample. An additional 32 women were excluded because of incomplete covariate data, resulting in a final analytic sample of 3265 women.

Following the demographic and health survey (DHS) methodological guidance, individual‐level weights were applied and adjusted for multilevel analysis. Although level‐specific weights are not directly available, previous studies indicate that using individual weights provides reasonable and unbiased estimates in multilevel models when higher‐level weights cannot be derived [23].

### 2.2. Study Variables and Measurements

#### 2.2.1. Outcome Variable

The outcome variables were self‐reported physician‐diagnosed depression, PHQ‐based depressive symptoms, and self‐reported physician‐diagnosed anxiety. Participants reported whether a healthcare provider had ever diagnosed them with depression or anxiety. PHQ‐based depressive symptoms reflected the current symptom status. Self‐reported diagnoses may be subject to recall bias and capture only diagnosed cases, potentially underestimating the true burden.

For the physician‐diagnosed depression and anxiety variables, participants who reported a lifetime diagnosis by a healthcare provider were coded as “1,” while those who reported no such diagnosis were coded as “0.” Similarly, PHQ‐based depressive symptoms were dichotomized, with individuals classified as “1” if they met the threshold for depressive symptoms and “0” if they did not.

It is important to note that the data reflect lifetime self‐reported diagnoses and rely on participants’ recall, which may introduce bias. Women who were never diagnosed by a healthcare provider are not captured, potentially underestimating the prevalence of depression and/or anxiety.

#### 2.2.2. Explanatory Variables

Based on the availability of data and evidence from the existing literature, a range of individual and contextual variables were included in the analysis. The individual‐level variables included age, sex of household head, educational status, occupation, media exposure, household wealth index, marital status, parity, pregnancy status, chronic illness, alcohol use, and health insurance coverage.

At the contextual (community) level, place of residence (urban or rural), region of residence, and perceived distance to a health facility were included to capture geographic and health service accessibility variations in the outcomes [24–28].

#### 2.2.3. Description of Explanatory Variables

PHQ‐based depressive symptoms (PHQ‐9): depressive symptoms were assessed using the PHQ‐9, which consists of nine items measuring the frequency of depressive symptoms over the past 2 weeks. Each item was scored from 0 (“not at all”) to 3 (“nearly every day”), with total scores ranging from 0 to 27. Standard severity categories include minimal (0–4), mild [5–9], moderate [10–14], moderately severe [15–19], and severe [20–27] depressive symptoms. For the purpose of this analysis, participants scoring ≥10 (moderate to severe depressive symptoms) were classified as having depressive symptoms (“1”), while those scoring <10 were classified as not having depressive symptoms (“0”).

### 2.3. Data Management and Statistical Analysis

Data handling and analysis were carried out using STATA version 17. Prior to the analysis, the presence, coding, and consistency of the self‐reported depression and/or anxiety variable in the LSDHS dataset were verified. All study variables were assessed for completeness, and records with missing values were excluded to ensure data integrity. The dataset was weighted to adjust for the complex survey design and nonproportional sampling across regions, ensuring accurate national estimates and valid standard errors. The final weighted sample comprised 3251 women of reproductive age.

Descriptive statistics were performed using weighted percentages to summarize the distribution of both individual and community‐level variables. Given the hierarchical structure of the DHS data where individuals are nested within households and clusters, multilevel logistic regression was employed to account for intra‐cluster correlation and contextual variability.

To examine factors associated with self‐reported physician‐diagnosed depression, PHQ‐based depressive symptoms, and self‐reported physician‐diagnosed anxiety, a multilevel logistic regression approach was used. Four models were constructed sequentially: the null model (baseline variance between clusters), model I (individual‐level variables), model II (community‐level variables), and model III (combined individual‐ and community‐level factors).

Model fit and comparison were assessed using several statistical criteria, including deviance (−2 log likelihood ratio [LR]), akaike information criterion (AIC), Bayesian information criterion (BIC), the intra‐class correlation coefficient (ICC), the median odds ratio (MOR), and the LR test. Multicollinearity was assessed using the variance inflation factor (VIF), and no evidence of multicollinearity was observed (mean VIF = 1.40; maximum VIF = 2.34) (Table 1). Statistical significance was determined at a *p*‐value < 0.05, and findings were reported as adjusted odds ratios (AORs) with 95% confidence intervals (CIs) [29].

**Table 1:** Assessment of multicollinearity among independent variables using the variance inflation factor (VIF) (weighted *n* = 3265).

Variable	VIF
Age	2.11
Sex of household	1.62
Marital status	1.56
Media exposure	1.42
Education	1.35
Occupation	1.35
Wealth	1.25
Parity	1.22
Currently pregnant	1.16
Chronic illness	1.06
Smoking cigarettes	1.05
Alcohol use	1.05
Money needed for treatment	1.04
Health insurance	1.02
Residence	1.01
Region	2.11
Distance to health facility	1.62

### 2.4. Ethical Considerations

This study is based on a publicly available and deidentified dataset obtained from the DHS Program. Ethical clearance was not required since there was no direct contact with the participants. Permission to access the 2023–2024 LSDHS dataset was granted by MEASURE DHS upon request.

## 3. Results

### 3.1. Background Characteristics of the Study Population

A total weighted sample of 3265 reproductive age women in Lesotho was included in this study. Most women were aged 20–29 years (33.5%) and lived in male‐headed households (57.6%). Over half were married (51.5%) and had media exposure (79.4%). Nearly 60% had at least secondary education, and 35.7% were employed. The majority were rural residents (56.5%), and 45.3% were in the rich wealth category. Multiparous women accounted for 37.4%, while currently pregnant women were few (3.1%). Chronic illness was reported in 10.6%, and most women lacked health insurance (90.1%). Depression and anxiety were notably higher among urban residents, women with media exposure, and those in the Maseru and Berea regions (Table 2).

**Table 2:** Descriptive characteristics of reproductive‐age women in Lesotho by sociodemographic and health‐related factors (weighted *n* = 3265).

Variables	Depression		Anxiety	
	No	Yes	No	Yes
Age				
15–19	596.17	52.49	590.78	57.88
20–29	922.89	115.30	927.05	111.14
30–39	784.46	94.87	798.08	81.25
40–49	585.71	113.73	614.32	85.12
Sex of household head				
Male	1675.76	200.90	1701.18	175.49
Female	1213.47	175.49	1229.06	159.91
Marital status				
Never in union	1046.62	128.01	1051.87	122.75
Married	1446.43	175.57	1450.57	171.43
Widowed/divorced	396.19	72.82	427.79	41.21
Media exposure				
No	588.19	65.21	593.05	60.36
Yes	2301.04	311.19	2337.19	275.04
Education				
No education	19.65	1.00	19.85	0.80
Primary	712.76	82.01	713.45	81.31
Secondary	1713.39	208.07	1747.57	173.89
Higher	443.44	85.33	449.37	79.40
Occupations				
No	1785.68	208.79	1806.89	187.59
Yes	1103.55	167.61	1123.35	147.81
Wealth				
Poor	927.26	80.63	929.51	78.38
Middle	562.37	65.22	579.65	47.94
Rich	1399.61	230.55	1421.08	209.07
Parity				
Nulliparous	950.88	103.94	952.89	101.93
Primiparous	678.87	83.08	676.24	85.71
Multiparous	1131.33	168.03	1171.88	127.49
Grand multiparous	128.14	21.34	129.22	20.27
Currently pregnant				
No	2807.94	364.51	2,843.41	329.04
Yes	81.29	11.89	86.83	6.36
Chronic illness				
No	2638.78	317.88	2665.48	291.18
Yes	250.45	58.53	264.76	44.22
Smoking cigarettes				
No	2822.18	366.19	2870.74	317.64
Yes	67.05	10.21	59.50	17.76
Alcohol use				
Not at all	2092.58	256.92	2115.69	233.81
1–5	698.57	100.64	714.32	84.89
6–10	49.41	13.04	52.01	10.45
11–25	27.93	3.78	27.41	4.29
Almost every day	20.74	22.75	20.80	1.96
Money needed for treatment				
Big problem	610.76	96.79	610.29	97.26
Not a big problem	2278.47	279.61	2319.95	238.14
Health Insurance				
No	2785.76	349.63	2819.26	316.13
Yes	103.4	26.77	110.98	19.26
Residency				
Urban	1256.62	213.46	1300.61	169.48
Rural	1632.61	162.94	1629.63	165.92
Region				
Butha‐Buthe	185.27	16.93	192.31	9.89
Leribe	538.05	47.69	553.69	32.05
Berea	408.23	90.68	397.16	101.75
Maseru	937.86	141.87	950.74	129.00
Mafeteng	181.39	26.87	188.02231	20.23
Mohale’s Hoek	137.53	11.13	142.71	5.95
Quthing	111.66	7.02	113.31	5.37
Qacha’s Nek	74.97	18.61	75.44	18.14
Mokhotlong	127.97	7.36	128.82	6.52
Thaba‐Tseka	186.29	8.22	188.04	6.48
Distance to health facility				
Big problem	724.64	89.26	1300.61	169.48
Not big problem	2164.59	287.13	1629.63	165.92

Abbreviations: AIC, akaike information criterion; AOR, adjusted odds ratio; BIC, Bayesian information criterion; CI, confidence interval; ICC, intraclass correlation coefficient; LLR, log‐likelihood ratio; MOR, median odds ratio; PCV, proportional change in variance; SE, standard error.

### 3.2. Prevalence of Depression and/or Anxiety

Among 3297 reproductive age women in Lesotho, 5.37% (*n* = 177) screened positive for depression based on PHQ scores, while 10.62% (*n* = 350) reported ever being told by a doctor or health care worker that they had depression. Additionally, 9.07% (*n* = 299) reported ever being told by a doctor or health care worker that they had anxiety.

### 3.3. Model Fitness and Factors Associated With Mental Health Outcomes

#### 3.3.1. Depression (Physician‐Diagnosed, Self‐Reported)

The ICC in the null model showed that 18.52% of the variation in depression was attributable to differences between clusters, with an MOR of 2.22, indicating higher odds of depression in higher‐risk clusters. Model III had the lowest deviance and was selected as the best‐fitting model.

In bivariate analysis, wealth, chronic illness, money needed for treatment, region, and distance to a health facility were significantly associated with depression.

In multivariable multilevel analysis, chronic illness (AOR = 1.97; 95% CI: 1.35, 2.87), living in Berea (AOR = 2.44; 95% CI: 1.39, 4.27) or Qacha’s Nek (AOR = 3.32; 95% CI: 1.83, 6.02), and reporting that treatment costs were not a big problem (AOR = 0.66; 95% CI: 0.49, 0.89) remained significant predictors of depression (Table 3).

**Table 3:** Multilevel multivariable logistic regression analysis of factors associated with physician‐diagnosed depression among reproductive age women in Lesotho (weighted *n* = 3265).

Variables	Model I AOR (95% CI)	Model II AOR (95% CI)	Model III AOR (95% CI)	
Age				
15–9	—	—	—	
20–29	1.16 (0.74, 1.82)	—	1.23 (0.79, 1.92)	
30–39	0.91 (0.52, 1.59)	—	0.98 (0.57, 1.71)	
40–49	1.08 (0.59, 1.96)	—	1.08 (0.60, 1.94)	
Wealth				
Poor	—	—	—	
Middle	1.32 (0.90, 1.94)	—	1.12 (0.76, 1.65)	
Rich	1.85 (1.27, 2.69)^a^	—	1.35 (0.89, 2.04)	
Chronic illness				
No	—	—	—	
Yes	1.83 (1.25, 2.67)	—	1.97 (1.35–2.87)^a^	
Money needed for treatment				
Big problem	—	—	—	
Not a big problem	0.60 (0.45, 0.80)^a^	—	0.66 (0.49, 0.89)^a^	
Residency				
Urban	—	—	—	
Rural	—	0.60 (0.45, 0.81)^a^	0.73 (0.52, 1.03)	
Region				
Butha‐Buthe	—	—	—	
Leribe	—	0.81 (0.44, 1.50)	0.75 (0.40, 1.41)	
Berea	—	2.45 (1.43, 4.20)^a^	2.44 (1.39, 4.27)^a^	
Maseru	—	1.66 (0.95, 2.88)	1.60 (0.91, 2.84)	
Mafeteng	—	1.75 (0.98, 3.15)	1.70 (0.93, 3.09)	
Mohale’s Hoek	—	0.86 (0.43, 1.68)	0.87 (0.44, 1.75)	
Quthing	—	0.64 (0.32, 1.29)	0.67 (0.33, 1.39)	
Qacha’s Nek	—	3.11 (1.75, 5.51)^a^	3.32 (1.83, 6.02)^a^	
Mokhotlong	—	0.68 (0.34, 1.35)	0.64 (1.83, 3.70)	
Thaba‐Tseka	—	0.52 (0.25, 1.07)	0.54 (0.25, 1.14)	
Distance to health facility				
Big problem	—	—	—	
Not big problem	—	0.72 (0.54, 0.96)^a^	0.85 (0.62,1.16)	

	Null model	Model I	Model II	Model III
Cluster level variance (SE)	0.18	0.18	0.13	0.14
ICC	18.52%	18.33%	8.84%	9.64%
PCV	Reference	1.34%	57.4%	53.01%
MOR	2.22	2.27	1.71	1.83
Deviance	2186.40	996.80	2107.97	2047.75
LLR	−1093.20	−1055.83	−1053.99	−1023.87
AIC	2190.40	2165.66	2133.97	2123.75
BIC	2202.60	2330.38	2330.38	2355.58

Abbreviations: AIC, akaike information criterion; AOR, adjusted odds ratio; BIC, Bayesian information criterion; CI, confidence interval; ICC, intraclass correlation coefficient; LLR, log‐likelihood ratio; MOR, median odds ratio; PCV, proportional change in variance; SE, standard error.

^a^Statistically significant at *p*‐value < 0.05.

#### 3.3.2. PHQ Depression

The ICC in the null model indicated that 8.62% of the variation in PHQ depression scores was attributable to differences between clusters, with an MOR of 1.70, suggesting moderate cluster‐level variation. Model III had the lowest deviance and was selected as the best‐fitting model.

In bivariate analysis, age 40–49, rich wealth, multiparity, grand multiparity, chronic illness, money needed for treatment, rural residence, Mohale’s Hoek, Mokhotlong, Thaba‐Tseka, and distance to health facility were significantly associated with PHQ depression.

In multivariable multilevel analysis, age 40–49 (AOR = 0.42; 95% CI: 0.20–0.90), multiparity (AOR = 2.20; 95% CI: 1.17–4.14), grand multiparity (AOR = 4.53; 95% CI: 1.87–10.99), chronic illness (AOR = 1.72; 95% CI: 1.05–2.81), not a big problem for treatment costs (AOR = 0.47; 95% CI: 0.33–0.69), Mohale’s Hoek (AOR = 0.32; 95% CI: 0.12–0.84), Mokhotlong (AOR = 0.18; 95% CI: 0.06–0.55), Thaba‐Tseka (AOR = 0.34; 95% CI: 0.14–0.87), and distance to the health facility (AOR = 1.57; 95% CI: 1.02–2.41) remained significant predictors of PHQ depression (Table 4).

**Table 4:** Multilevel multivariable logistic regression analysis of factors associated with PHQ‐based depressive symptoms among reproductive age women in Lesotho (weighted *n* = 3265).

Variables	Model I AOR (95% CI)	Model II AOR (95% CI)	Model III AOR (95% CI)	
Age				
15–19	—	—	—	
20–29	0.69 (0.39, 1.22)	—	0.70 (0.39, 1.24)	
30–39	0.59 (0.29, 1.17)	—	0.61 (0.31, 1.23)	
40–49	0.43 (0.20, 0.92)^a^	—	0.42 (0.20, 0.90)^a^	
Wealth				
Poor	—	—	—	
Middle	1.34 (0.83, 2.17)	—	1.06 (0.65–1.73)	
Rich	1.77 (1.13, 2.77)^a^	—	1.23 (0.74–2.05)	
Parity				
Nulliparous	—	—	—	
Primiparous	1.31 (0.74, 2.34)	—	1.26 (0.71–2.25)	
Multiparous	2.30 (1.22, 2.32)^a^	—	2.20 (1.17–4.14)^a^	
Grand multiparous	2.46 (1.85, 2.76)	—	4.53 (1.87–10.99)^a^	
Chronic illness				
No	—	—	—	
Yes	1.59 (0.97, 2.59)	—	1.72 (1.05–2.81)^a^	
Money needed for treatment				
Big problem	—	—	—	
Not a big problem	0.54 (0.38, 0.78)^a^	—	0.47 (0.33–0.69)^a^	
Urban	—	—	—	
Rural	—	0.77 (0.55, 1.09)^a^	0.86 (0.57–1.29)	
Region				
Butha‐Buthe	—	—	—	
Leribe	—	1.05 (0.57, 1.94)	0.97 (0.52–1.83)	
Berea	—	1.29 (0.71, 2.35)	1.25 (0.67–2.32)	
Maseru	—	0.96 (0.52, 1.78)	0.93 (0.49–1.75)	
Mafeteng	—	0.96 (0.49, 1.87)	0.91 (0.46–1.84)	
Mohale’s Hoek	—	0.36 (0.14, 0.92)^a^	0.32 (0.12–0.84)^a^	
Quthing	—	1.05 (0.53, 2.06)	0.99 (0.49–2.00)	
Qacha’s Nek	—	0.94 (0.46, 1.91)	0.87 (0.41–1.82)	
Mokhotlong	—	0.22 (0.07, 0.65)^a^	0.18 (0.06–0.55)^a^	
Thaba‐Tseka	—	0.35 (1.14, 0.85)^a^	0.34 (0.14–0.87)^a^	
Distance to health facility				
Big problem	—	—	—	
Not big problem	—	1.12 (0.76, 1.64)	1.57 (1.02–2.41)^a^	
	**Null model**	**Model I**	**Model II**	**Model III**
Cluster level variance (SE)	0.31	0.28	0.13	0.18
ICC	8.62%	7.74%	3.71%	5.20%
PCV	Reference	10.91%	59.16%	41.83%
MOR	1.70	1.65	1.40	1.50
Deviance	1375.84	1323.07	1339.37	1287.92
LLR	−687.92	−661.54	−669.68	−643.96
AIC	1379.85	1377.07	1365.37	1363.92
BIC	1392.05	1541.79	1444.68	1595.75

Abbreviations: AIC, akaike information criterion; AOR, adjusted odds ratio; BIC, Bayesian information criterion; CI, confidence interval; ICC, intraclass correlation coefficient; LLR, log‐likelihood ratio; MOR, median odds ratio; PCV, proportional change in variance; SE, standard error.

^a^Statistically significant at *p*‐value < 0.05.

#### 3.3.3. Anxiety (Physician‐Diagnosed, Self‐Reported)

The ICC in the null model indicated that 20.85% of the variation in anxiety scores was attributable to differences between clusters, with an MOR of 2.26, indicating a notable cluster‐level variation. Model III had the lowest deviance and was selected as the best‐fitting model.

In bivariate analysis, media exposure, rich wealth, chronic illness, cigarette smoking, money needed for treatment, and region (Berea, Maseru, Mafeteng, and Qacha’s Nek) were significantly associated with anxiety.

In multivariable multilevel analysis, media exposure (AOR = 0.71; 95% CI: 0.50–1.00), chronic illness (AOR = 2.11; 95% CI: 1.38–3.22), cigarette smoking (AOR = 2.34; 95% CI: 1.76–3.31), and region (Berea [AOR = 4.59; 95% CI: 2.43–8.68], Maseru [AOR = 2.56; 95% CI: 1.33–4.92], Mafeteng [AOR = 2.18; 95% CI: 1.08–4.36], and Qacha’s Nek [AOR = 5.07; 95% CI: 2.58–9.94]) remained significant predictors of anxiety (Table 5).

**Table 5:** Multilevel multivariable logistic regression analysis of factors associated with anxiety among reproductive age women in Lesotho (weighted *n* = 3265).

Variables	Model I AOR (95% CI)	Model II AOR (95% CI)	Model III AOR (95% CI)	
Media exposure				
No	—	—	—	
Yes	0.80 (0.57, 1.13)	—	0.71 (0.50, 1.00)^a^	
Wealth				
Poor	—	—	—	
Middle	1.15 (0.76, 1.75)	—	1.02 (0.67, 1.56)	
Rich	1.88 (1.25, 2.83)^a^	—	1.46 (0.93, 2.27)	
Chronic illness				
No	—	—	—	
Yes	1.85 (1.21, 2.83)^a^	—	2.11 (1.38, 3.22)^a^	
Smoking cigarettes				
No	—	—	—	
Yes	1.16 (0.75, 1.79)^a^	—	2.34 (1.76, 3.31)^a^	
Money needed for treatment				
Big problem	—	—	—	
Not a big problem	0.71 (0.52, 0.97)^a^	—	0.73 (0.52–1.01)	
Region				
Butha‐Buthe	—	—	—	
Leribe	—	1.10 (0.54, 2.24)	1.07 (0.52–2.20)	
Berea	—	4.52 (2.43, 8.41)^a^	4.59 (2.43–8.68)^a^	
Maseru	—	2.66 (1.41, 5.04)^a^	2.56 (1.33–4.92)^a^	
Mafeteng	—	2.20 (1.12, 4.36)^a^	2.18 (1.08–4.36)^a^	
Mohale’s Hoek	—	0.77 (0.33, 1.80)	0.76 (0.32–1.80)	
Quthing	—	0.86 (0.38, 1.92)	0.85 (0.37–1.95)	
Qacha’s Nek	—	5.00 (2.59, 9.64)^a^	5.07 (2.58–9.94)^a^	
Mokhotlong	—	0.92 (0.42, 2.03)	0.82 (0.36–1.84)	
Thaba‐Tseka	—	0.65 (0.28, 1.51)	0.57 (0.24–1.34)	
	**Null model**	**Model I**	**Model II**	**Model III**
Cluster level variance (SE)	0.21	0.21	0.10	0.11
ICC	20.85%	21.38%	10.25%	10.96%
PCV	Reference	2.45%	51.0%	53.4%
MOR	2.26	2.28	1.72	1.84
Deviance	1958.65	1955.13	1889.14	1888.99
LLR	−977.32	−950.56	−931.50	−906.57
AIC	1960.65	1955.13	1888.99	1889.14
BIC	1970.85	2119.85	1968.31	2120.97

Abbreviations: AIC, akaike information criterion; AOR, adjusted odds ratio; BIC, Bayesian information criterion; CI, confidence interval; ICC, intraclass correlation coefficient; LLR, log‐likelihood ratio; MOR, median odds ratio; PCV, proportional change in variance; SE, standard error.

^a^Statistically significant at *p*‐value < 0.05.

## 4. Discussion

This study assessed the prevalence and determinants of depression and anxiety among reproductive age women in Lesotho using nationally representative survey data. The findings revealed that 10.62% of women reported physician‐diagnosed depression, 5.37% had depressive symptoms based on the PHQ screening, and 9.07% reported anxiety. Depression and anxiety are among the most prevalent mental health conditions worldwide and represent major public health challenges because of their negative impact on individuals’ social functioning and quality of life.

The prevalence observed in this study is higher than the prevalence reported in a study conducted using the Kenya DHS, which found that 3.84% of adults experienced depression and/or anxiety. The difference may be explained by variations in the study populations, measurement approaches, and sociocultural contexts. Differences in healthcare access, stigma toward mental illness, and the use of screening versus diagnostic approaches may also contribute to the variation in prevalence across countries [30].

Regional differences in depression and anxiety were observed in this study. Significant community‐level variation was identified among reproductive age women in Lesotho, with those residing in Berea, Maseru, and Qacha’s Nek exhibiting higher odds of mental health outcomes compared to women in the Butha‐Buthe region. This finding indicates that geographic location plays an important role in shaping mental health outcomes. The presence of between‐cluster variation, as indicated by the ICC and MOR, further supports the influence of contextual factors beyond individual‐level characteristics. Such geographic disparities in mental health outcomes have also been documented in previous studies, suggesting that differences in socioeconomic conditions, access to healthcare services, and availability of mental health resources may contribute to the unequal distribution of mental health disorders across populations. These contextual differences likely explain the observed regional variation in this study [24, 31].

Chronic illness was strongly associated with depression, PHQ‐based depression, and anxiety in this study, consistent with the global evidence that persistent physical conditions exacerbate emotional distress. Women with chronic diseases had higher odds of mental health problems compared with those without such conditions. A study conducted in Greece similarly reported higher levels of depression and anxiety among patients with chronic diseases [32]. Likewise, a longitudinal study from China found that individuals with chronic conditions had an increased risk of developing depression, with the risk rising as the number of chronic diseases increased [33]. Chronic illness may contribute to psychological distress through ongoing symptoms, functional limitations, and financial strain, alongside biological mechanisms such as dysregulation of stress‐response systems. These findings highlight the need to integrate mental health screening and counseling into chronic disease management services.

Financial accessibility to healthcare was also associated with depression outcomes in this study. Women who reported that obtaining money for medical treatment was not a major problem had lower odds of depression and depressive symptoms compared with those who faced financial barriers. A similar finding was reported in a study conducted in Bangladesh, which indicated that economic hardship and a lack of financial resources were strongly associated with depressive symptoms among women. Financial stress may increase psychological distress and limit access to healthcare services, which can worsen both physical and mental health outcomes [34].

Age was also associated with depressive symptoms in this study. Women aged 40–49 years had lower odds of depressive symptoms compared with those of adolescents aged 15–19 years. Younger women may experience higher levels of psychological stress due to social pressures, economic instability, and life transitions. Previous epidemiological studies have also indicated that depressive and anxiety disorders often occur earlier in life and are influenced by social and environmental stressors. A nationally representative study from the United States found that the prevalence of major depressive episodes during childhood (under age 13) was 10.03% and during adolescence (ages 13–18) was 14.12%, with women being particularly impacted by the early onset [35].

Behavioral factors were associated with anxiety in the present study. Cigarette smoking was associated with a higher odds of anxiety among women. Smoking is frequently used as a coping strategy for stress and emotional distress; however, nicotine dependence may worsen anxiety symptoms and contribute to long‐term psychological problems. Studies examining mental–physical comorbidity have also shown that smoking and other behavioral risk factors are associated with both depression and anxiety [36].

Media exposure was found to be protective against anxiety in this study. Women who reported media exposure had lower odds of anxiety compared with those without media exposure. Access to information through media platforms may improve mental health literacy and increase awareness of mental health conditions and available healthcare services. Improved awareness may encourage individuals to seek professional support when experiencing psychological distress [37].

## 5. Limitations of the Study

This cross‐sectional study cannot establish causal relationships between the identified factors and mental health outcomes. Depression and anxiety based on physician diagnosis were self‐reported, which may be subject to recall bias and likely underestimate the true burden, as only diagnosed cases were captured. Although PHQ‐based depressive symptoms were included to assess the current mental health status, anxiety was measured only through self‐reported diagnosis due to the unavailability of GAD data in the dataset. Future studies should incorporate standardized screening tools such as PHQ and GAD simultaneously to provide a more comprehensive assessment of current mental health conditions.

## 6. Conclusions

In Lesotho, a notable proportion of women experience mental health problems, with 10.62% reporting self‐reported physician‐diagnosed depression, 5.37% meeting the criteria for PHQ‐based depressive symptoms, and 9.07% reporting self‐reported physician‐diagnosed anxiety. Chronic illness was consistently associated with all outcomes, while financial barriers, parity, age, smoking, media exposure, and regional factors showed outcome‐specific associations. These findings suggest the need for integrated and targeted mental health interventions, particularly within primary healthcare and chronic disease services, to address the burden among women.

**Nomenclature**
AIC: Akaike information criterion
AOR: Adjusted odds ratio
BIC: Bayesian information criterion
CI: Confidence interval
DHS: Demographic and health survey
EA: Enumeration area
ICC: Intraclass correlation coefficient
IR: Individual recode
LMICs: Low‐ and middle‐income countries
LSDHS: Lesotho demographic and health survey
MLM: Multilevel modeling
MOR: Median odds ratio
SD: Standard deviation
STATA: Data analysis and statistical software (Stata Corp)
VIF: Variance inflation factor
WHO: World Health Organization.

## Author Contributions

Helen Lamesgin Endalew was involved in conceiving the idea, study design, data analysis and interpretation, writing the manuscript, and managing the overall progress of the study. Astewil Moges Bazezew, Eniyew Assimie Alemu, Enyew Getaneh Mekonen, Berihun Agegn Mengistie, Gebreeyesus Abera Zeleke Desalegn Getachew Ayele, Amlaku Nigusie Yirsaw, Getie Mihret Aragaw, Alemken Eyayu Abuhay, Degsew Ewunetie Anteneh, and Gebeyehu Lakew were involved in study design, data analysis, and in revising the manuscript.

## Funding

No funding was received for this manuscript.

## Disclosure

The final manuscript was read and approved by all the authors.

## Ethics Statement

The study used publicly available, deidentified data from the 2023/2024 Demographic and Health Survey (DHS). Ethical clearance for the collection of DHS data was obtained by the DHS Program from the relevant national ethics committees. Access to the dataset for this study was granted by the DHS Program, and all analyses were conducted in accordance with the DHS data use agreement, ensuring the confidentiality and privacy of respondents.

## Consent

The authors have nothing to report.

## Conflicts of Interest

The authors declare no conflicts of interest.

## Data Availability

The dataset used in this study is publicly available through the Demographic and Health Surveys (DHS) Program. Access to the 2023–2024 Lesotho Demographic and Health Survey (LSDHS) data was granted upon request via the DHS website (https://dhsprogram.com/). All data used in this analysis were deidentified, and no personally identifiable information was included.
